# Territory Differences in Adaptation to Heat among Persons Aged 65 Years and Over in Spain (1983–2018)

**DOI:** 10.3390/ijerph20054168

**Published:** 2023-02-25

**Authors:** Miguel Ángel Navas-Martín, José Antonio López-Bueno, María Soledad Ascaso-Sánchez, Fernando Follos, José Manuel Vellón, Isidro Juan Mirón, María Yolanda Luna, Gerardo Sánchez-Martínez, Julio Díaz, Cristina Linares

**Affiliations:** 1 National School of Public Health, Carlos III Institute of Health, 28029 Madrid, Spain; 2 Doctorate Program in Biomedical Sciences and Public Health, National University of Distance Education, 28015 Madrid, Spain; 3 Tdot Soluciones Sostenibles, SL. Ferrol, 15401 A Coruña, Spain; 4 Regional Health Authority of Castile La Mancha, 45500 Torrijos, Spain; 5 State Meteorological Agency, 28071 Madrid, Spain; 6 The UNEP DTU Partnership, 2100 Copenhagen, Denmark

**Keywords:** adaptation, MMT, age, elderly, rural, nonurban, urban, mortality, health

## Abstract

Climate change is currently regarded as the greatest global threat to human health, and its health-related consequences take different forms according to age, sex, socioeconomic level, and type of territory. The aim of this study is to ascertain the differences in vulnerability and the heat-adaptation process through the minimum mortality temperature (MMT) among the Spanish population aged ≥65 years by territorial classification. A retrospective, longitudinal, ecological time-series study, using provincial data on daily mortality and maximum daily temperature across the period 1983–2018, was performed, differentiating between urban and nonurban populations. The MMTs in the study period were higher for the ≥65-year age group in urban provinces, with a mean value of 29.6 °C (95%CI 29.2–30.0) versus 28.1 °C (95%CI 27.7–28.5) in nonurban provinces. This difference was statistically significant (*p* < 0.05). In terms of adaptation levels, higher average values were obtained for nonurban areas, with values of 0.12 (95%CI −0.13–0.37), than for urban areas, with values of 0.09 (95%CI −0.27–0.45), though this difference was not statistically significant (*p* < 0.05). These findings may contribute to better planning by making it possible to implement more specific public health prevention plans. Lastly, they highlight the need to conduct studies on heat-adaptation processes, taking into account various differential factors, such as age and territory.

## 1. Introduction

Climate change continues to wreak havoc in many regions of the world and is now currently regarded as the greatest global threat to human health [1,2]. The Intergovernmental Panel on Climate Change (IPCC) estimates that global temperatures will rise by 1.5 °C above preindustrial levels over the next decade. One of the many effects of climate change is the increase in average temperatures and heatwaves, which are more intense, more frequent, and longer [3,4]. Therefore, human activity has brought about changes in the climate, causing serious harm to nature and persons, especially in the most vulnerable groups [5].

The risk of heat exposure is a global issue, with population exposure to heatwaves increasing by 57% on average between 2010 and 2019 compared to the previous decade. This increase puts vulnerable populations, such as older people, young children, and those with chronic health conditions, at high risk of heat-related morbidity and mortality. Additionally, global warming has been associated with an estimated temperature-related mortality increase in most regions, causing an average of 15.1 additional deaths per million inhabitants per decade. Heat exposure not only has direct health impacts, but it also undermines people’s livelihoods and social determinants of health by reducing labor capacity. In Europe, where there are aging populations, urbanization, and a high prevalence of chronic diseases, the risk of heat-related health problems has increased across all regions, with a relative increase of 9.8% observed in central Europe. Although northern Europe is the most vulnerable region, all areas are affected [6].

The rise in temperatures has led to growing concern about the association between temperature, morbidity, and mortality. High temperatures increase the risk of cardiovascular and respiratory disorders, heat stroke, exacerbation of kidney and neurodegenerative diseases, and even death [7,8,9], with the risk varying according to age, sex, socioeconomic level, and type of territory. Several studies in different continents have reported greater vulnerability to heat among women and the elderly [10,11,12,13,14], with the elderly being considered the principal susceptible group affected by nonoptimal temperatures and the ensuing conclusion that more attention should be paid to climates with high, or even moderate, temperatures [15].

Several research studies have determined that the impact of nonoptimal temperatures on mortality can be influenced by various demographic characteristics, including age. There is a consistent association between elderly people and increased vulnerability to the risks of heat exposure [16,17,18]. Individuals over the age of 65 are highly susceptible to temperature-induced fatalities, primarily due to changes in their thermoregulatory system. Specifically, their sweat response and thirst sensation are reduced. Moreover, older adults are typically less physically fit and have more illnesses and disabilities, which further increase their vulnerability to heat-related morbidity and mortality [19].

The factors that make a person vulnerable to heat stress are unique to each individual. When heat waves occur, certain groups of people are more at risk of experiencing negative health outcomes, including mortality and morbidity. This susceptibility is often seen in individuals who have impaired physiological and behavioral responses to heat, which can be attributed to their advanced age [20]. Nonetheless, advanced age is not the only factor that contributes to heat susceptibility in older individuals. Other contributing factors are physiological, socioeconomic, and behavioral issues. Physiological factors include conditions such as low fitness levels, cardiovascular and renal insufficiencies, pre-existing chronic health conditions, psychogeriatric and neurocognitive disorders, and certain medications, among others. Socioeconomic factors include social isolation and financial concerns related to energy costs. Additionally, older housing, the perception that heat does not pose a health risk, and reluctance to change behavior are other contributing factors that increase the risk of heat events [21].

The world’s aging population is increasing both in absolute numbers and as a percentage of the total population. By 2050, it is projected that 16% of the global population will be aged 65 or above, with Europe and Northern America having the largest proportion of older persons in 2022. The projected growth rates of populations in different regions will result in a significant shift in the regional distribution of the global population by 2050, with one in every four persons in Europe and Northern America being aged 65 years or over [22].

The European Union is concerned about demographic shifts. Population growth trends are not the same in urban and rural areas, as the average annual growth rate of the rural population is declining in all world regions and is expected to decrease even further to about −2% by the year 2025 in Europe. Rural areas in Southern Europe are particularly affected by aging and depopulation, with a predicted decrease of 18.5% in the rural population from 2015 to 2030 [23].

The problem of the rural–urban gradient becomes more critical with the aging of the population, in particular, southern and Mediterranean countries stand out for having a high proportion of elderly people residing in rural areas. The population over 65 years old exceeds 20% and even a quarter of the total in some regions. Spain and Portugal are among the countries with the highest rural aging rates. The aging population in rural areas presents challenges, such as an increased need for personal assistance due to chronic pathologies, leading to greater dependency compared to urban areas [24].

The population lives in urban areas, in particular, have significant concentrations of elderly residents and are the dwelling places for 43.2% of the older populace, where the impacts of heat on health are exacerbated by the characteristics of buildings and infrastructures, the effects of heat islands, and air pollution [5,25,26,27,28], with these areas, thus being hotter than rural areas [8,29,30]. In an increasingly more urbanized world, the number of people living in settings affected by urban heat islands is expected to increase in the future [29,31]. In a large proportion of towns and cities, health centers, such as hospitals, nursing homes, and social housing, are located in areas that experience the urban heat-island effect, thereby increasing the exposure of vulnerable groups [8,32]. Furthermore, the inequalities between urban and rural populations in terms of socioeconomic status, lifestyle, or access to health care can make for different vulnerabilities to environmental stress [33].

In certain measures, populations that are acclimatized tend to be technically and behaviorally adapted to their local climate. That said, however, heat-related mortality continues to occur because adaptation is not complete, coupled with the fact that there are differences between regions [34] and a lack of information about how quickly the population is adapting to the increase in temperatures caused by climate change [35,36].

A good indicator for measuring a given population’s vulnerability and capacity for adaptation to heat is the minimum mortality temperature (MMT). The temperature-mortality relationship is graphically represented by a U-shaped curve, where the minimum coincides with the temperature at which the risk of mortality is lowest, reflecting the optimal and most comfortable temperature for human beings [1]. The MMT is influenced by numerous factors [34], including age group [37] and type of territory [38].

The vulnerability is the risk to which a system is vulnerable to negative consequences of climate change, including climate unpredictability and extremes [39]. Heat vulnerability is determined by both environmental (such as the frequency of heat waves) and individual factors. Factors such as age, pre-existing diseases, the level of hydration, or housing conditions, among others [40]. In contrast, adaptation is the process of adjusting human systems to the effects of the current or predicted climate in order to minimize harm or take advantage of advantageous chances [41]. Through a variety of targeted measures, adaptation primarily tries to moderate the negative consequences of unavoidable climate change [42].

In extreme heat, it is crucial for older adults to engage in adaptive behaviors to prevent the onset of heat-related illnesses. Many older adults spend a significant amount of time at home, which is vital to their well-being, but older homes may not provide adequate thermal comfort. To maintain a comfortable temperature, adaptive measures such as moving to a cooler location, wearing light clothing, increasing fluid intake, taking cool showers or baths, reducing physical activity, or using cooling devices can be helpful. However, the willingness and ability to adopt these behaviors depend on individuals’ heat perception and health knowledge. Overall, older adults need to understand the importance of adaptive behavior during heat episodes and make informed decisions about which measures they can adopt to protect themselves from heat stress [21].

There are a number of studies investigating the relationship between mortality and temperature and the risk of vulnerability in the elderly [9,43,44,45] and vulnerability to heat between rural and urban populations [46,47]. In contrast, there are fewer studies on heat adaptation [48] and fewer in the elderly or between rural and urban populations, and even fewer still that quantify adaptation by age and territory simultaneously.

This study thus sought to use the time trend in the MMT to ascertain differences in the Spanish elderly population’s adaptation to heat by type of territory. To this end, a retrospective, longitudinal, ecological time-series study was conducted, using the mortality rates of the population aged ≥ 65 years and maximum daily temperatures from 1983 to 2018.

## 2. Materials and Methods

**Figure 1:**
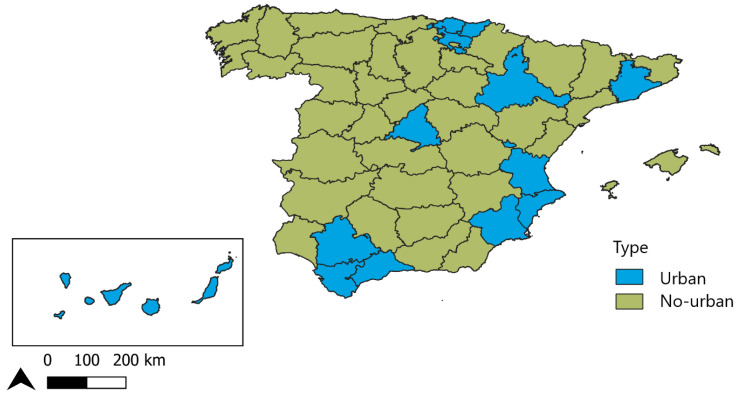
Map of urban and nonurban (intermediate and rural) provinces in Spain, 2015.

To achieve the above objective, we conducted a retrospective, longitudinal, ecological study in Spain across the period 1983–2018. The basis for performing a comparative analysis by territory was the classification proposed by Eurostat in 2015 to define types of areas as urban (i.e., predominantly urban) or nonurban (i.e., corresponding to intermediate and predominantly rural regions) [38,49] (Figure 1).

We used daily mortality data, coded by all causes of death (ICD 10: A00-R99), covering the ≥65-year age group, aggregated by town. These mortality data corresponded to towns of over 10,000 inhabitants. Daily mortality and population data, furnished by the National Statistics Institute (Instituto Nacional de Estadística (INE)) under a Microdata Access Agreement, were used to calculate the relevant rates per 100,000 population.

For meteorological information on maximum daily temperatures, we used data registered at reference observatories in each province. These data were supplied by the State Meteorological Agency (Agencia Estatal de Meteorología (AEMET)).

The following were discarded: any record lacking mortality and temperature data, and any annual series in respect of which more than 10% of valid records were missing.

### 2.1. Calculation of MMT

Applying a previously described deterministic method [10,50,51], MMTs were calculated for each year and province, using daily mortality and maximum daily temperature grouped into intervals of two degrees Celsius. We then fitted a cubic or quadratic regression model of mortality with temperature, selecting the MMT values that were statistically significant (*p* < 0.05). In cases where the MMT was not significant, estimates were made to calculate the remaining MMTs. The calculation of MMTs by estimation is performed by using the average of the maximum daily temperature observed under the 5th percentile of mortality. This is a methodology that has been used in previous studies to complement MMT values not yielded by a cubic or quadratic fit. In any case where MMTs were not obtained by estimation, the MMTs for that year were discarded and deemed to be null.

### 2.2. Determination of Heat-Adaptation Levels

Based on the annual MMTs, we calculated the line of fit over time for each province. The slope determines the MMT’s rate of variation over time in °C/decade (MMT Variation). The same was then done with the annual mean of the maximum daily temperature, obtaining TMAX’s rate of variation in °C/decade (TMAX Rise). The adaptation level was obtained as the difference between the respective rates of variation, in °C/decade, for MMT (MMT Variation) and TMAX (TMAX Rise), namely, Adaptation Level = MMT Variation − TMAX Rise.

If the adaptation level is >0, this would indicate that the MMT has risen more rapidly than the TMAX, and could thus be considered a process of adaptation to heat. Once the MMTs had been calculated for each province, the provinces were grouped according to the definition of the territory, namely, urban and nonurban.

### 2.3. Data Analysis

To ascertain the trend in the MMT across the study period, a linear regression model was fitted for MMT by year, according to the type of territory. To represent the provinces according to the territories that did or did not show adaptation to heat, we drew up a scatter plot of MMT with respect to TMAX, indicating the area of the adaptation zone. Data processing was performed using the IBM SPSS Statistics version 28, R version 4.0.2, STATA BE-Basic Edition version 17, and Excel 2019 (with the Power Query add-in) computer software programs.

## 3. Results

A total of 92.4% (*n* = 1596) of MMTs were obtained: 71.1% (*n* = 1229) by cubic fit, 12.6% (*n* = 218) by estimation-based fit, and 8.6% (*n* = 149) by quadratic fit, with 7.6% (*n* = 132) classified as null. The MMTs in the study period were higher for the elderly group aged ≥65 years in urban provinces (Table 1), with a mean value of 29.6 °C (95%CI 29.2–30.0) versus 28.1 °C (95%CI 27.7–28.5) in nonurban provinces (Table 2). This difference was statistically significant (*p* < 0.05).

The MMT rate of variation (°C/decade) among the elderly population recorded a higher average value in nonurban areas (Table 2), 0.48 °C/decade (95%CI 0.2–0.7), than in urban areas (Table 1), 0.38 °C/decade (95%CI 0.0–0.7), though this difference was not statistically significant. In terms of adaptation levels, a higher average value was obtained for nonurban areas (Table 2) than for urban areas (Table 1), with figures of 0.12 (95%CI −0.13–0.37) versus 0.09 (95%CI −0.27–0.45), respectively. Here again, the difference was not statistically significant.

**Figure 2:**
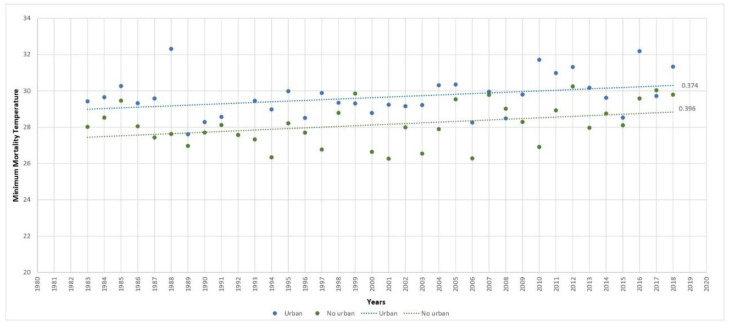
Trend in minimum mortality temperature (MMT) by year and type of territory in Spain (1983–2018).

With respect to the MMT time trend for the population aged ≥ 65 years by territory (Figure 2), this was higher in nonurban than urban areas, with values of 0.40 (°C/decade) versus 0.37 (°C/decade), respectively.

**Figure 3:**
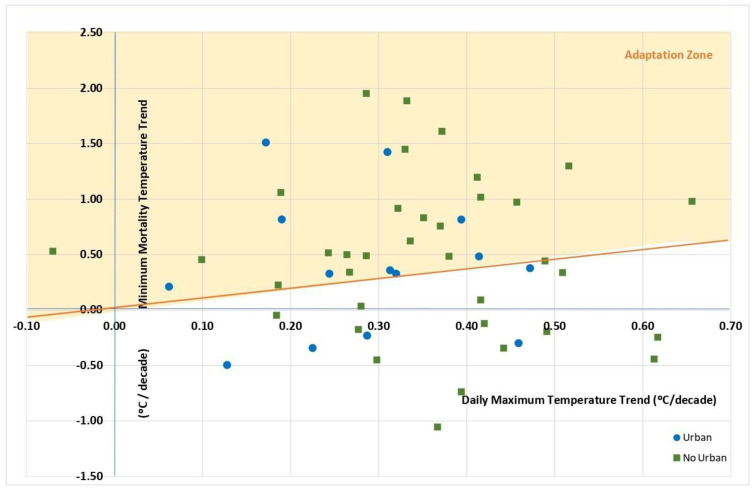
Scatter plot of the minimum mortality temperature with respect to variations in maximum daily temperatures, by territory in Spain (1983–2018). The shaded area indicates provinces showing adaptation to heat.

Lastly, according to the scatter plot of the variations in MMT with respect to those in TMAX (Figure 3), both urban (9 out of 14) and nonurban provinces (22 out of 36) were more represented in the adaptation zone, though the territorial difference was only 3.2 percentage points higher in urban than in nonurban areas.

## 4. Discussion

While vulnerability to climate change differs according to population differences, giving rise to different adaptative responses among the respective social and demographic groups [52], there are few studies that currently analyze the process of population adaptation to the impacts of climate change [53]. This study made it possible to ascertain the level of adaptation by type of territory by analyzing the MMT trend in the population aged ≥65 years across the period 1983–2018.

The results show that the average MMT value was higher in urban (29.6 °C) than nonurban provinces (28 °C). Hence, if the MMT were taken as an indicator of vulnerability to heat, this would mean that the elderly population aged ≥65 years was more vulnerable to heat in nonurban than in urban provinces. In this connection, a study in China on urban–rural inequalities reported that persons aged ≥65 years showed a higher relative risk for heat in rural than in urban areas [54].

Although previous studies undertaken in Spain show the general population of nonurban regions to be less vulnerable to heat [47], different factors such as socioeconomic level and health-service access, among others, generate urban–rural inequalities in the population, and these in turn may then generate different patterns of vulnerability to the impact of heat [54]. The time trend in the rate of variation in the MMT for the home provinces of the populations aged ≥ 65 years was upward for the country as a whole, and was higher than the variation in the increase in the maximum daily temperature. Adaptation levels were thus positive and showed adaptation to heat. Even so, the level was slightly higher in nonurban areas. There were no statistically significant differences (*p* < 0.05) by territory, either across time or at a provincial level. In general, adaptation to heat is occurring among the elderly population, in urban and nonurban areas alike, since the upward shifts in the value of the MMT have been shown to displace the entire temperature-mortality curve [51,55].

While the heat-adaptation level was higher in rural (0.12) than in urban areas (0.09), the margin was minimal, with just 0.03 points of difference (°C/decade) and, in addition, was not statistically significant. These differences are related to the way in which temperature is related to mortality can vary by region [56], and the general population’s capacity for acclimatization and adaptation differs, as between regions [57], in the case of the elderly, the elderly in Spanish provinces showed similar levels of adaptation.

While little is known about the patterns [58] and pace of adaptation [36], it is nevertheless clear that when the rate of variation of the MMT is higher than that of the maximum daily temperature, the population shows adaptation [38]. Adaptation can be attributed to a number of factors, ranging from physiological adaptation [19] to other factors of a social, cultural, health, economic, and/or infrastructural nature [51]. There are many determinants related to heat adaptation, including access to financial resources, counseling, electricity, age, occupation, and the availability of alert systems, among others [59].

Socioeconomic, cultural, general, and environmental conditions influence the social determinants associated with the mortality of elderly persons. Housing, stress, financial situation as dictated by income level, education, ethnicity, and/or territory (rural–urban), among other factors, determine social inequalities in health, which in turn play a key role in mortality [60]. It is therefore necessary to ascertain which factors specific to the elderly may affect their adaptation to heat. More in-depth studies are called for in order to be able to identify some of these factors, which include housing characteristics and health-service access, among others.

## 5. Limitations

This study has a number of limitations, the first of which is its very nature, in that being an ecological study, the results cannot be extrapolated at an individual level [61].

Secondly, there is the limitation of the concept of rurality itself, since there is no universally accepted definition of the term [47]. It was for this reason that we opted for a classification that would allow for grouping and making comparisons by reference to territory. Similarly, the data had to be aggregated in order to be able to perform the analysis, bearing in mind that population groups are not homogeneous and that subpopulational differences may therefore exist.

Lastly, there is the limitation of the methodology for calculating MMTs, since there are different approaches [62], and this, in turn, limits comparisons with other studies [34]. Added to the fact that there is no single universal methodology, there are the geographical, climatic, and socioeconomic characteristics of each region that influence and determine the heterogeneity of results [63]. That said, however, the study uses the same methodology as previous studies undertaken in the same study setting.

There is also a degree of geographical disparity [58,64], which renders comparison between regions difficult, in view of the differences caused by geographical, climatic, and socioeconomic factors [63]. The lack of sufficient evidence to indicate how these differences may affect the factors that influence adaptation means that more specific, individualized studies should be undertaken in order to enable such factors to be identified in greater detail [38,58].

Despite these limitations, this study applied the same methodology for the calculation of every single province. Furthermore, this study will allow for comparison, not only with other studies conducted in the past, but also with future studies having the same geographical scope.

## 6. Conclusions

The MMTs for the population aged ≥ 65 years were higher in urban than in nonurban provinces. Nevertheless, the trend in the rate of variation and level of adaptation was higher in nonurban areas. It can therefore be concluded that nonurban areas showed better adaptation than urban areas, though these differences were not statistically significant. These findings may serve to understand the differences in adaptation to heat, according to geographical area, among the population ≥65 years. Lastly, this study highlights the need, both to conduct observational epidemiology studies for the purpose of planning personalized public health prevention actions, and to take into account the different differential factors, such as age and territory, which intervene in heat-adaptation processes.

## Figures and Tables

**Table 1:** Relationship of variables, by urban province, between minimum mortality temperature (MMT) in persons aged 65 years or over, showing MMT mean, TMAX mean, trend by decade in TMAX, variations in MMT, and adaptation levels. * *p* < 0.05.

Province	MMT Mean	TMAX Mean (°C)	TMAX Rise (°C/Decade)	MMT Variation (°C/Decade)	Adaptation Level (MMT Variation-Tax Rise)
Arabia	28.2	17.4	0.459	−0.299	−0.758
Alicante	30.2	23.5	0.190	0.817	0.627 *
Barcelona	26.9	20.6	0.414	0.483	0.069 *
Cadiz	28.2	21.7	0.287	−0.230	−0.517
Gipuzkoa	26.3	16.6	0.244	0.328	0.084
Madrid	29.2	20.2	0.394	0.816	0.422 *
Malaga	31.5	23.5	0.320	0.327	0.007
Murcia	30.3	22.4	0.172	1.510	1.338 *
Palmas, Las	29.4	24.3	0.128	−0.495	−0.623
S.C. Tenerife	30.1	24.7	0.225	−0.342	−0.567
Seville	34.0	25.6	0.310	1.425	1.115 *
Valencia	31.0	22.9	0.313	0.359	0.046
Bizkaia (Biscay)	29.1	19.7	0.062	0.210	0.148
Zaragoza	30.0	21.3	0.472	0.377	−0.095
(Spain)	29.6	21.74	0.29	0.38	0.09

**Table 2:** Relationship of variables, by nonurban province, between minimum mortality temperature (MMT) in persons aged 65 years or over, MMT mean, TMAX mean, trend by decade in TMAX, variations in MMT, and adaptation levels. * *p* < 0.05.

Province	MMT Mean	TMAX Mean (°C)	TMAX Rise (°C/Decade)	MMT Variation (°C/Decade)	Adaptation Level (MMT Variation-Tmax Rise)
Albacete	30.4	21	0.509	0.337	−0.172
Almería	31.3	23.4	−0.070	0.531	0.601
Avila	23.1	17.2	0.394	−0.737	−1.131
Badajoz	32.8	24	0.286	0.490	0.204
Balearic Isles	28.6	22	0.330	1.449	1.119 *
Burgos	27.4	16.8	0.372	1.611	1.239
Cáceres	29.7	22.1	0.336	0.623	0.287
Castellón	29.9	22.5	0.370	0.757	0.387
Ciudad Real	29.5	22	0.267	0.341	0.074
Cordoba	34.3	25.4	0.332	1.887	1.555 *
Corunna	24.7	18	0.351	0.832	0.481
Cuenca	26.1	19.6	0.617	−0.245	−0.862
Girona	29.5	21.1	0.656	0.980	0.324
Granada	31.7	22.6	0.416	1.018	0.602 *
Guadalajara	26.4	20.5	0.367	−1.054	−1.421
Huelva	30.4	24.1	0.322	0.916	0.594
Huesca	27.8	19.8	0.489	0.442	−0.047
Jaén	30.2	21.8	0.516	1.299	0.783 *
León	26.4	16.9	0.243	0.516	0.273
Lleida	30.3	21.7	0.264	0.499	0.235
Rioja, La	27.5	19.8	0.416	0.091	−0.325
Lugo	27.9	17.8	0.189	1.060	0.871
Navarre	27.2	18.6	0.442	−0.344	−0.786
Ourense	31.4	21.6	0.457	0.973	0.516
Asturias	25.3	17.5	0.184	−0.047	−0.231
Palencia	24.0	16.8	0.286	1.953	1.667
Pontevedra	26.4	19.1	0.099	0.455	0.356
Salamanca	27.4	19	0.613	−0.442	−1.055
Cantabria	26.6	18.7	0.277	−0.175	−0.452
Segovia	23.8	18.1	0.298	−0.450	−0.748
Soria	24.3	17.3	0.280	0.035	−0.245
Tarragona	28.8	21.3	0.380	0.484	0.104
Teruel	23.8	19.9	0.420	−0.122 *	−0.542
Toledo	30.2	22.4	0.412	1.197	0.785 *
Valladolid	26.5	17.8	0.186	0.225 *	0.039
Zamora	25.9	19.2	0.491	−0.194	−0.685
(Spain)	28	20.21	0.36	0.48	0.12

## Data Availability

Not applicable.
